# Multiple local σ-aromaticity of nonagermanide clusters†
†Electronic supplementary information (ESI) available. See DOI: 10.1039/c9sc00929a


**DOI:** 10.1039/c9sc00929a

**Published:** 2019-04-29

**Authors:** Nikolay V. Tkachenko, Alexander I. Boldyrev

**Affiliations:** a Department of Chemistry and Biochemistry , Utah State University , 0300 Old Main Hill , Logan , UT 84322-0300 , USA . Email: a.i.boldyrev@usu.edu

## Abstract

Nonagermanide clusters are widely used in inorganic synthesis and are actively studied by experimentalists and theoreticians. However, chemical bonding of such versatile species is still not completely understood. In our work we deciphered a bonding pattern for various experimentally obtained nonagermanide species. We localized the electron density *via* the AdNDP algorithm for the model structures ([Ge_9_]^4–^, [Ge_9_{P(NH_2_)_2_}_3_]^–^, Cu[Ge_9_{P(NH_2_)_2_}_3_] and Cu(NHC)[Ge_9_{P(NH_2_)_2_}_3_]) and obtained a simple and chemically intuitive bonding pattern which can explain the variety of active sites and the existence of both *D*_3h_ and *C*_4v_ geometries for such clusters. Moreover, the [Ge_9_]^4–^ core is found to be a unique example of an inorganic Zintl cluster with multiple local σ-aromaticity.

## Abstract 2

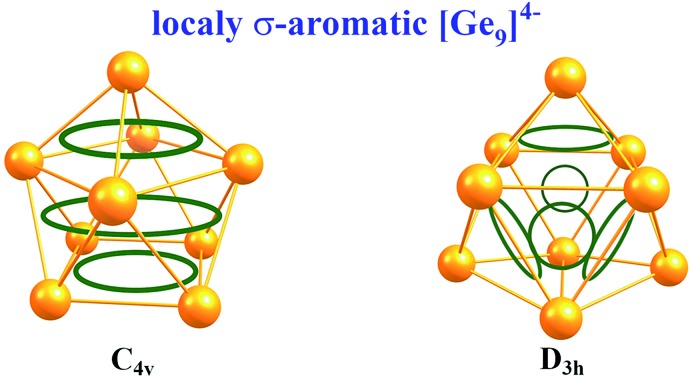
A new chemical bonding picture of various nonagermanide species were presented. The stability of Ge_9_ fragments could be well described *via* the concept of multiple local σ-aromaticity.

## Introduction

The concept of aromaticity has long gone beyond the classical Hückel organic structures.^1^,^2^ Today, the terms π- and σ-aromaticity are well used for a variety of compounds, including complex inorganic structures.^3^ In these cases, the delocalized bonding well describes the formation of highly symmetric stable compounds. Numerous planar boron clusters,^4^–^7^ metal clusters,^3^,^7^–^13^ and metallabenzenes^14^–^18^ fit well into the concept of aromaticity. Along with π- and σ-aromaticity, multiple local π aromaticity is found to be a useful tool to describe complex conjugate systems such as graphene.^19^–^21^ This concept has been accepted up to a point. In our work we showed that the concept of multiple local σ-aromaticity could be applied in chemistry. It should be noted that the analysis of completely delocalized canonical MOs cannot lead to a reliable picture of chemical bonding in such clusters,^22^,^23^ since there is a mixture of electron lone pairs (an element that does not participate in chemical bonding interactions) with bonding elements of the cluster. Thus, to analyse aromaticity, firstly we need to localize all classical elements (such as 1c–2e lone pairs and 2c–2e Lewis bonds). The adaptive natural density partitioning algorithm (AdNDP) can be very useful for this purpose.

Homoatomic clusters of type [M_9_]^z–^ (M = Si, Ge, Sn, and Pb) have been investigated both experimentally and theoretically.^24^,^25^ The structures of most such clusters confirm the predictions from Wade's rules.^26^,^27^ Interestingly, the expectation of the geometry of [M_9_]^4–^ species with 2n+4 (*n* = 9) skeletal electrons leads us to a *nido* nine-vertex polyhedron (*C*_4v_ capped square antiprism).

**Fig. 1:**
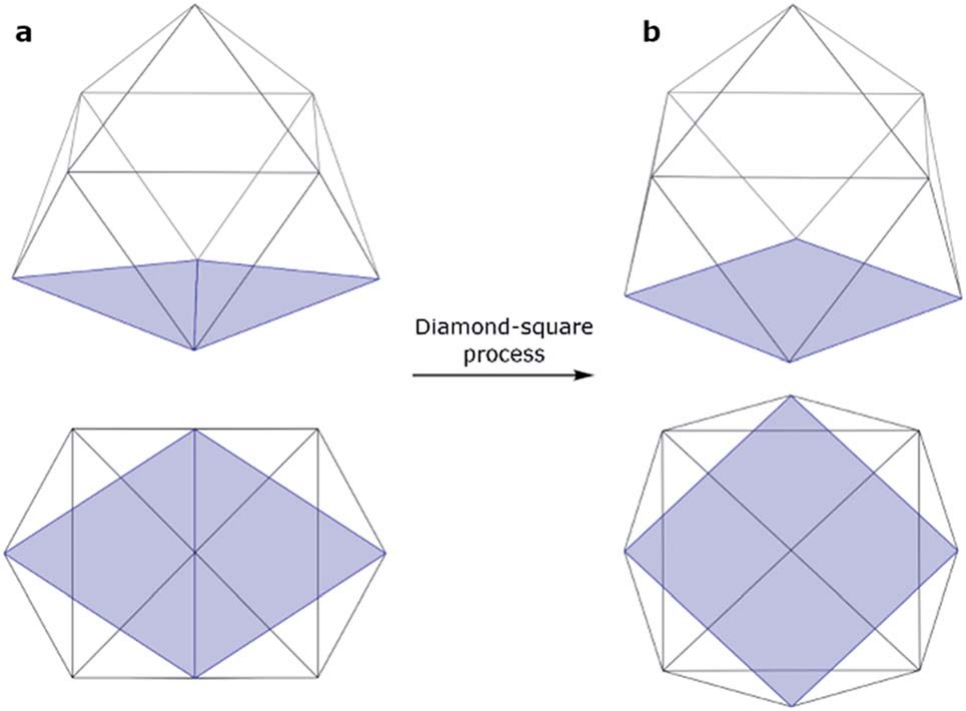
Side and top views of a (a) tricapped trigonal prism (*D*_3h_ symmetry) and (b) capped square antiprism (*C*_4v_ symmetry).

An isolated [Ge_9_]^4–^ cluster has been experimentally obtained in the solid state in the form of alkali metal salts (K_4_Ge_9_,^28^ Cs_4_Ge_9_ and Rb_4_Ge_9_ (ref. 29)). The geometry of these clusters (except Rb_4_Ge_9_) was found by X-ray crystallography and it was slightly distorted from the ideal *C*_4v_ capped square antiprism, confirming Wade's rules.^24^ However, various DFT calculations (Table 1) suggest that the global minimum structure for [Ge_9_]^4–^ is a tricapped trigonal prism (*D*_3h_ symmetry).^23^,^30^ In turn, the *C*_4v_ symmetric structure has one imaginary frequency. A similar picture could be found, for instance, in a biphenyl system (twisted geometry in the gaseous phase and completely planar in the solid state). *D*_3h_ and *C*_4v_ structures transform from one to another through the diamond square process (Fig. 1).^31^,^32^

**Table 1:** Relative energies with ZPE corrections (kcal mol^–1^) of *D*_3h_ and *C*_4v_ [Ge_9_]^4–^ structures at various levels of theory

Structure	PBE0			B3LYP		
	LANL2DZ	Aug-cc-pvdz	Aug-cc-pvtz	LANDL2DZ	Aug-cc-pvdz	Aug-cc-pvtz
*D*_3h_ [Ge_9_]^4–^	0.0	0.0	0.0	0.0	0.0	0.0
*C*_4v_ [Ge_9_]^4–^	0.0	1.6^*a*^	10.2^*a*^	0.0	0.4^a^	9.1^*a*^

^*a*^Structure is a first order stationary point.

Although Wade's rules can give us a prediction of the cluster structure, we still do not have a comprehensive picture of the chemical bonding for such species. In this paper, we presented a new description of bonding in [Ge_9_]^4–^ clusters, based on the local σ-aromaticity concept. The obtained bonding pattern explains both the presence of active sites and the existence of *C*_4v_ and *D*_3h_ geometries for these compounds.

## Computational methods

All structures were optimized at the PBE0/aug-cc-pvdz^33^,^34^ level of theory. Frequency calculations were performed using the harmonic approximation. To understand the chemical bonding pattern, matrix representation of the first order reduced density operator in the NAO basis set was analysed by the adaptive natural density partitioning (AdNDP) algorithm^35^ as implemented in the AdNDP 2.0 code^36^ at the PBE0/cc-pvdz level of theory. In the AdNDP method, we followed general ideas of the NBO analysis proposed by Weinhold.^37^ It represents a chemical bonding structure in terms of *n*c–2e bonds following the concept of electron pairs and 2c–2e classical bonds. To assess the aromaticity of each aromatic fragment, nucleus-independent chemical shift (NICS)^38^,^39^ calculations were performed at the PBE0/aug-cc-pvtz level. The ChemCraft 1.8 software was used to visualize chemical bonding patterns and geometries of the investigated compounds.

## Results and discussion

**Fig. 2:**
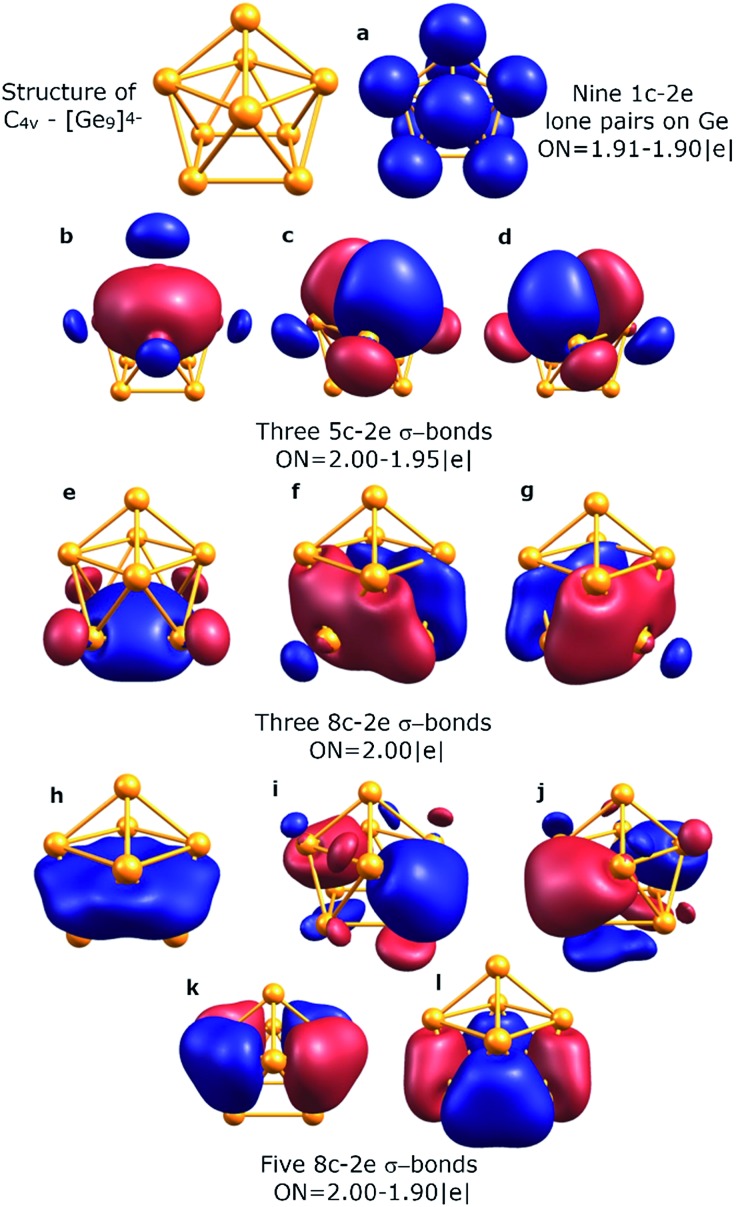
Overall chemical bonding picture obtained for the *C*_4v_ capped square antiprism [Ge_9_]^4–^ cluster. ON denotes the occupation number (equal to 2.00 |e| in an ideal case). Lines between atoms help in visualization and do not represent 2c–2e bonds here and elsewhere.

To understand the stability of both *D*_3h_ and *C*_4v_ isomers, we conducted AdNDP analysis of the first order reduced NAO density matrix for *C*_4v_ and *D*_3h_ [Ge_9_]^4–^ clusters. We found that the *C*_4v_ structure has nine classical Lewis lone pairs on germanium atoms with occupation numbers (ONs) 1.91–1.90 |e|. The remaining electron density forms eleven delocalized σ-bonds with ON = 2.00–1.90 |e| (Fig. 2). These delocalized σ-type fragments bind three areas of the germanium cluster. The first area is the cap of the square antiprism (Fig. 2b–d) and consists of three 5c–2e bonds with ON = 2.00–1.95 |e|. The shape of these elements as well as the number of electrons (6 electrons fit the 4n+2 Hückel's rule) indicates σ-aromaticity in the Ge_5_ cap fragment. Next three 8c–2e σ-bonds with ON = 2.00 |e| bind the bottom square fragment (Fig. 2e–g). The contribution of this Ge_4_ fragment into the 8c–2e bonds is ∼99.0% for the bond shown in Fig. 2e and ∼81.9% for bonds shown in Fig. 2f and g. The whole Ge_8_ square antiprism fragment is bound by five 8c–2e σ-bonds with ON = 2.00–1.90 |e|. The shapes of 8c–2e and 5c–2e bonds (one bond without a nodal plane, two bonds with one nodal plane, *etc.*) and 4n+2 electrons (*n* = 1,2) on each area render these fragments σ-aromatic. Therefore, the stability of the *C*_4v_ structure can be explained by the existence of three σ-aromatic areas: the Ge_5_ cap fragment, Ge_4_ bottom square fragment and Ge_8_ square antiprism fragment.

**Fig. 3:**
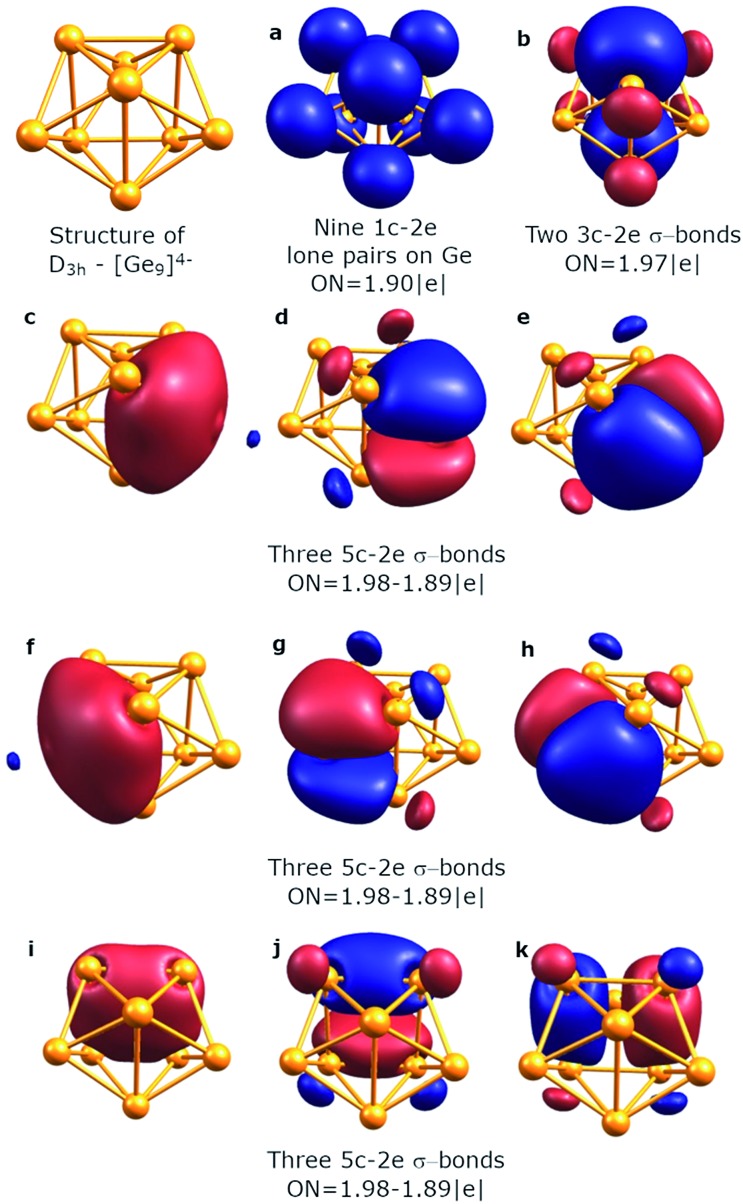
Overall chemical bonding picture obtained for the *D*_3h_ tricapped trigonal prism [Ge_9_]^4–^ cluster.

An equally interesting picture of chemical bonding was found for the *D*_3h_ [Ge_9_]^4–^ cluster (Fig. 3). According to AdNDP analysis, the 40 valence electrons in [Ge_9_]^4–^ can be localized into nine classical 1c–2e lone pairs on Ge atoms, two 3c–2e sigma bonds with ON = 1.97 |e|, and nine delocalized 5c–2e bonds. These nine 5c–2e bonds with ON = 1.98–1.89 |e| are responsible for binding interactions inside each Ge_5_ cap fragment (rectangular pyramids built on the sides of a trigonal prism). Three σ-bonds per cap constitute σ-aromaticity. It should be mentioned that the 3c–2e bonds could be also considered σ-aromatic since the number of electrons fits well in Hückel's rule (*n* = 0). Thus, the chemical bonding pattern for the *D*_3h_ [Ge_9_]^4–^ cluster includes nine lone pairs, two 3c–2e σ-aromatic bonds and three symmetric Ge_5_ σ-aromatic areas with 5c–2e σ-bonds, which explains the stability and the variety of active sites for such a structure.

**Fig. 4:**
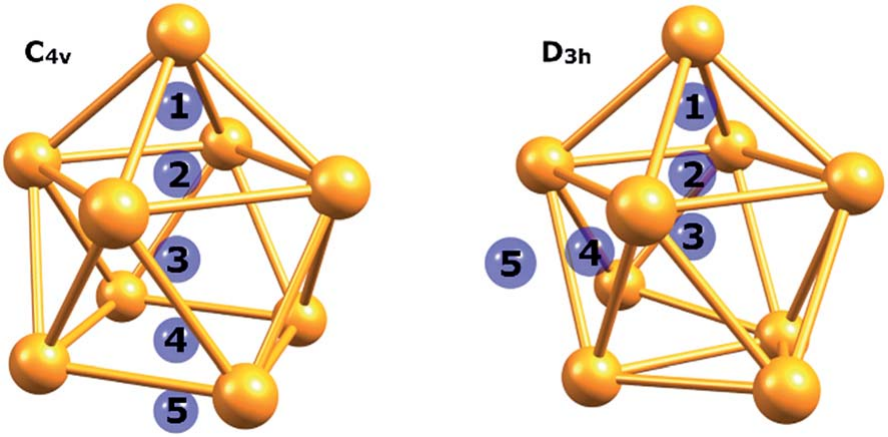
Points that were chosen for the NICS indices calculation.

To assess local σ-aromaticity in *D*_3h_ and *C*_4v_ structures, we calculated NICS_zz_ and NICS_iso_ indices at different points of the clusters (Fig. 4). The coordinates of chosen points can be found in the ESI (Table S2†). Significantly negative values of NICS indices at the centers of aromatic fragments (points 1, 3, and 4 for *C*_4v_ and points 1 and 4 for the *D*_3h_ structure) corroborate our local σ-aromatic description of the bonding pattern for such clusters (Table 2).

**Table 2:** NICS_zz_ and NICS_iso_ indices calculated at chosen points for *D*_3h_ and *C*_4v_ [Ge_9_]^4–^ clusters

Point	*C*_4v_ [Ge_9_]^4–^		*D*_3h_ [Ge_9_]^4–^	
	NICS_zz_	NICS_iso_	NICS_zz_	NICS_iso_
1	–152.42	–85.91	–62.91	–50.81
2	–102.32	–94.10	–90.53	–67.92
3	–99.76	–90.68	–90.47	–65.89
4	–97.46	–78.02	–87.71	–55.38
5	–55.43	–26.92	–68.37	–22.68

It should be noted that the described bonding pattern is not unique to germanium clusters and could be found in other isoelectronic species. To show that, we performed AdNDP analysis for [Si_9_]^4–^ and [Sn_9_]^4–^ clusters. The results are similar and can be found in the ESI (Fig. S4–S6†).

**Fig. 5:**
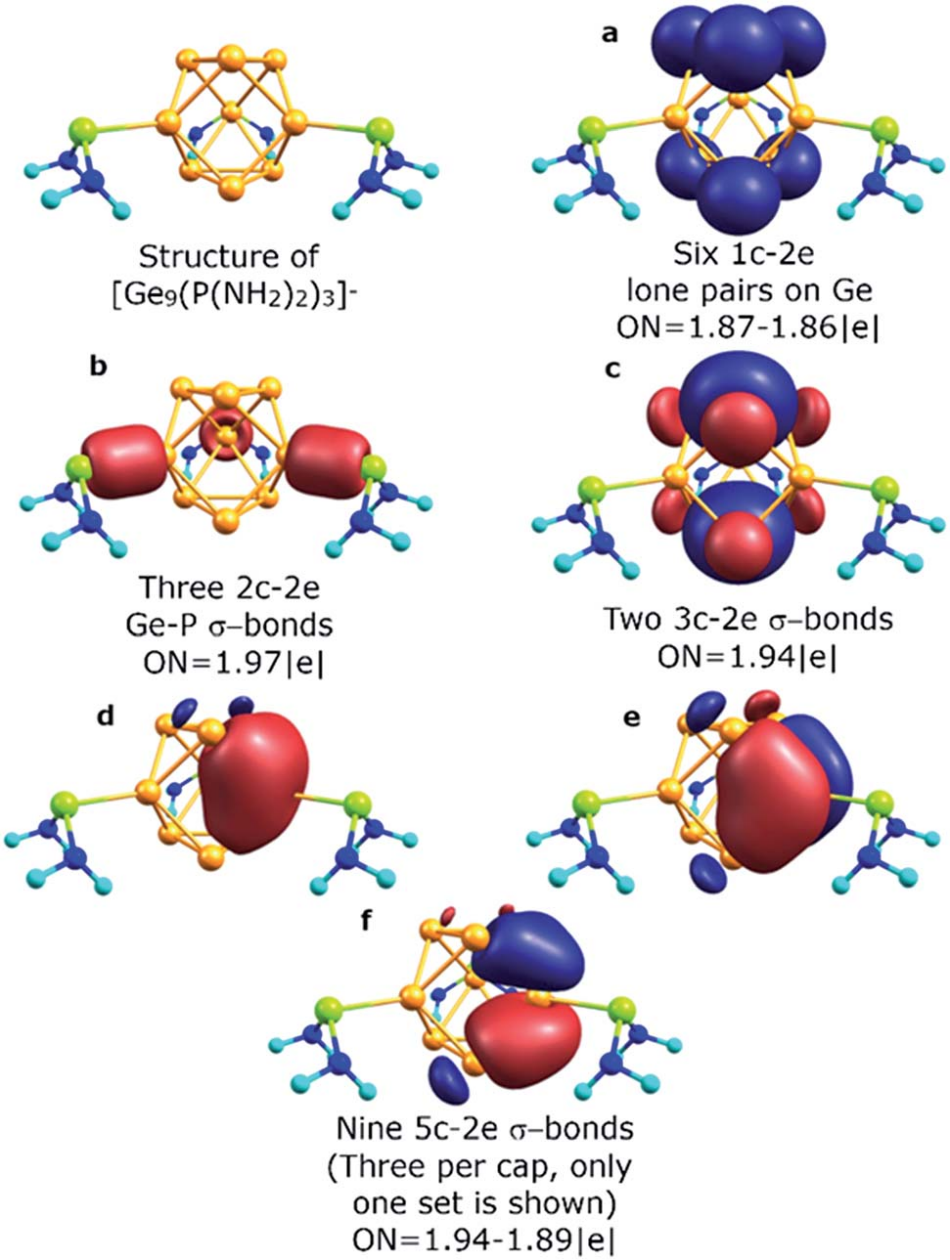
Chemical bonding picture of the core Ge_9_ fragment obtained for the [Ge_9_{P(NH_2_)_2_}_3_]^–^ cluster.

The described chemical bonding pattern also could be found in other synthesized germanium clusters. Various derivatized nonagermanide systems were reported,^40^–^51^ including mono-, di- and tri-substituted species. Of all the series, threefold phosphine-functionalized nonagermanide clusters, synthesized in 2018 by Fässler and coworkers, are of particular interest due to their high reactivity and ease of synthesis.^51^ In these clusters, the geometry of the core Ge_9_ fragment is found to be slightly distorted from that of the tricapped *D*_3h_ symmetric trigonal prism. In our model calculations of the [Ge_9_{P(N^*i*^Pr_2_)_2_}_3_]^–^ cluster, we attached three P(NH_2_)_2_^+^ substituents to the three capping Ge atoms. The results are shown in Fig. 5. We found that the bonding pattern of the core Ge_9_ fragment remains the same as that of the *D*_3h_-[Ge_9_]^4–^ cluster. The main difference in bonding is that three peripheral 1c–2e lone pairs on capping Ge atoms participate in the formation of new two-center classical bonds with phosphorus (ON = 1.97 |e|). The occupancy of the nine 5c–2e bonds, which are responsible for the local σ-aromaticity, remains almost the same (Fig. 5d–f). The –P(NH_2_)_2_ substituents exhibit a classical bonding pattern with 1c–2e lone pairs on P (ON = 1.94 |e|) and N atoms (ON = 1.91 |e|) and 2c–2e σ N–H (ON = 1.99 |e|) and σ N–P (ON = 1.99 |e|) bonds (Fig. S1†).

Previously, an intriguing reactivity was found for such threefold phosphine-functionalized nonagermanide clusters.^51^ The reaction of [Ge_9_{P(N^*i*^Pr_2_)_2_}_3_]^–^ and NHC^Dipp^CuCl leads to the formation of a (NHC^Dipp^Cu)[Ge_9_{P(N^*i*^Pr_2_)_2_}_3_] compound, containing the *D*_3h_ symmetric Ge_9_ fragment. For understanding the bonding pattern within the copper containing structure, we started our analysis from the model molecule – (Cu)[Ge_9_{P(NH_2_)_2_}_3_]. The results are shown in Fig. S2.† We found that the Cu atom keeps five 1c–2e d-type lone pairs with an occupation number of 1.99–1.96 |e|. The bonding pattern of the [Ge_9_{P(NH_2_)_2_}_3_]^–^ part remains almost the same, except one 3c–2e σ-aromatic fragment on the top of the trigonal prism that transforms into a 4c–2e Ge_3_Cu σ-bond with ON = 1.97 |e|. The contribution of the Ge_3_ triangle fragment into the 4c–2e bond is found to be ∼83.7%. Even though this model molecule is a good object for studying bonding patterns, the optimized geometry noticeably differs from the experimentally obtained structure. For instance, the Ge–Ge distance in the upper triangle of the trigonal prism is 3.17 Å (experimental value is 2.87 Å). That is why we decided to include the NHC ligand attached to the Cu atom, which models the NHC^Dipp^ ligand.

**Fig. 6:**
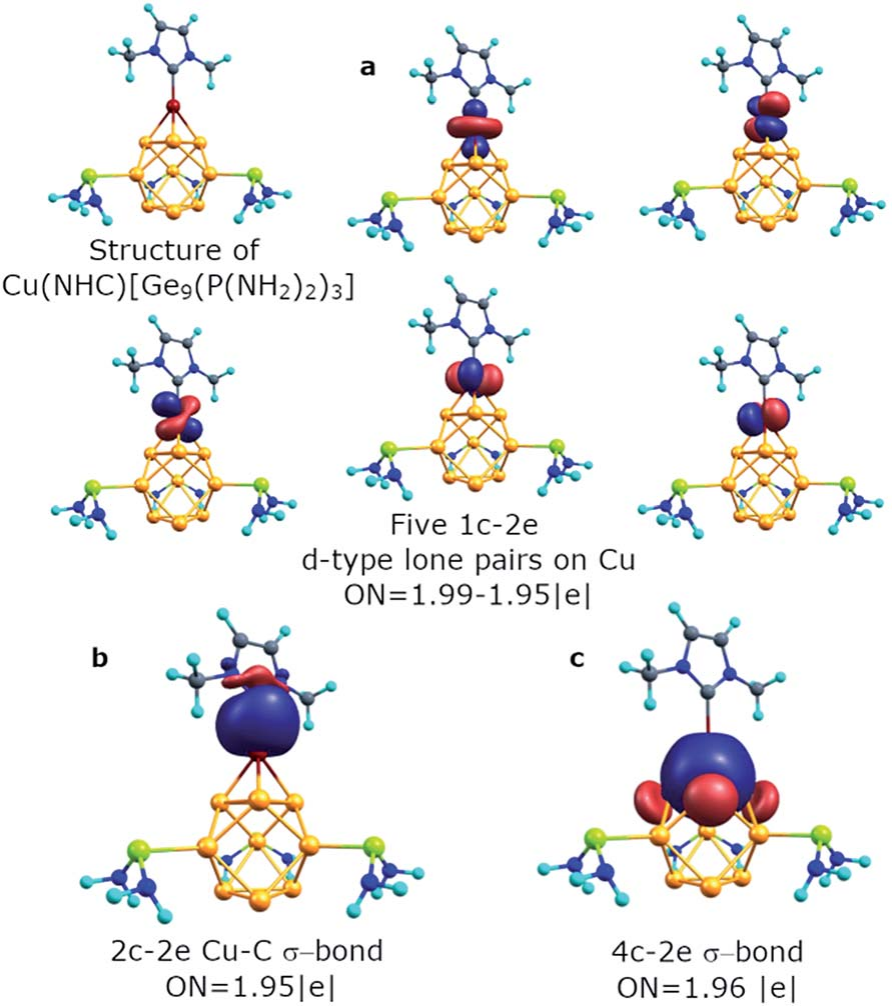
Chemical bonding of the Cu atom in the Cu(NHC)[Ge_9_{P(NH_2_)_2_}_3_]^–^ cluster.

The geometry of the optimized structure with the NHC ligand attached to the Cu atom corroborates with the experimental data. So, the Ge–Ge distance in the upper triangle and the distance between capped germanium and germanium in the upper triangle of the prism is 2.93 Å and 2.50 Å, respectively (experimental data are 2.87 Å and 2.50 Å, respectively). It should be mentioned that the attachment of the second [Ge_9_{P(NH_2_)_2_}_3_]^–^ fragment to the (Cu)[Ge_9_{P(NH_2_)_2_}_3_] molecule also leads to Ge–Ge distances similar to the experimental ones (Table S1,† {[Ge_9_{P(NH_2_)_2_}_3_]Cu[Ge_9_{P(NH_2_)_2_}_3_]}^–^ structure). By conducting AdNDP analysis we found that the bonding pattern of the core Ge_9_ fragment remains the same (Fig. S3†). It consists of six s-type 1c–2e lone pairs on Ge atoms, three local σ-aromatic Ge_5_ fragments, one 3c–2e σ-aromatic bond at the bottom, and one 4c–2e CuGe_4_ σ-bond at the top of the prism. The binding interactions of the Cu atom are illustrated in Fig. 6. The new 2c–2e σ Cu–C bond with ON = 1.95 |e| appears due to the attachment of the NHC ligand. The contribution of the carbon atom into the Cu–C bond is assessed to be ∼85.3%, indicating the donor–acceptor type of the sigma bond.

## Conclusions

In summary, we performed a chemical bonding analysis for five nonagermanide species. The stability of both *D*_3h_ and *C*_4v_ structures of the Ge_9_^4–^ cluster could be well described *via* the concept of multiple local σ-aromaticity. We are not aware of such a chemical bonding in the literature. On the basis of AdNDP analysis we can separate the electron density in locally aromatic regions: the Ge_5_ cap, Ge_8_ antiprism, and Ge_4_ square fragments for *C*_4v_ [Ge_9_]^4–^, and then three Ge_5_ caps, and two 3c–2e triangular fragments for *D*_3h_ [Ge_9_]^4–^. We found a similar bonding pattern for the core Ge_9_ fragment for previously synthesized trisubstituted [Ge_9_{P(NR_2_)_2_}_3_] clusters. The –P(NH_2_)_2_ groups are bound with the tricapped trigonal prism core *via* classical 2c–2e Ge–P σ-bonds.

A variety of active sites are well described with the obtained bonding picture. Thus, it was found that the copper atom in Cu[Ge_9_{P(NH_2_)_2_}_3_] and Cu(NHC)[Ge_9_{P(NH_2_)_2_}_3_] clusters interacts with the 3c–2e σ-bond of the Ge_9_ core to form a 4c–2e bond. Therefore, the described picture is indeed in good agreement with the experimental reactivity of such compounds. The same chemical bonding was also found in isoelectronic [Si_9_]^4–^ and [Sn_9_]^4–^ clusters. We hope that the new obtained bonding pattern with locally σ-aromatic fragments could be an important step to build a coherent bonding structure of Ge-family clusters and congener species. It will also help in the development and understanding of chemical bonding for complicated cases in inorganic chemistry.

## Conflicts of interest

There are no conflicts to declare.

## Supplementary Material

Supplementary information
