# Supplementary material for: Multiple local σ-aromaticity of nonagermanide clusters
Source: Chem Sci. 2019 Apr 29;10(22):5761–5. doi: 10.1039/c9sc00929a (PMC6568043; doi:10.1039/c9sc00929a)
Supplement: Supplementary file 1 [file SC-010-C9SC00929A-s001.pdf]

Electronic Supplementary Material (ESI) for Chemical Science.
This journal is © The Royal Society of Chemistry 2019

Multiple Local σ-Aromaticity of the Nonagermanide Clusters

Nikolay V. Tkachenko and Alexander I. Boldyrev*
Department of Chemistry and Biochemistry
Utah State University
Logan, Utah 84322-0300

**e-mail: a.i.boldyrev@usu.edu*

KEYWORDS: multiple σ-aromaticity, Zintl anions, chemical bonding, germanium, reactivity

Supporting Information

Contents

1. Complete chemical bonding pattern of selected species S2
2. Optimized geometries of investigated molecules.. S7
3. Coordinates of chosen points for NICS calculation.. S20

1. Complete chemical bonding pattern of selected species.

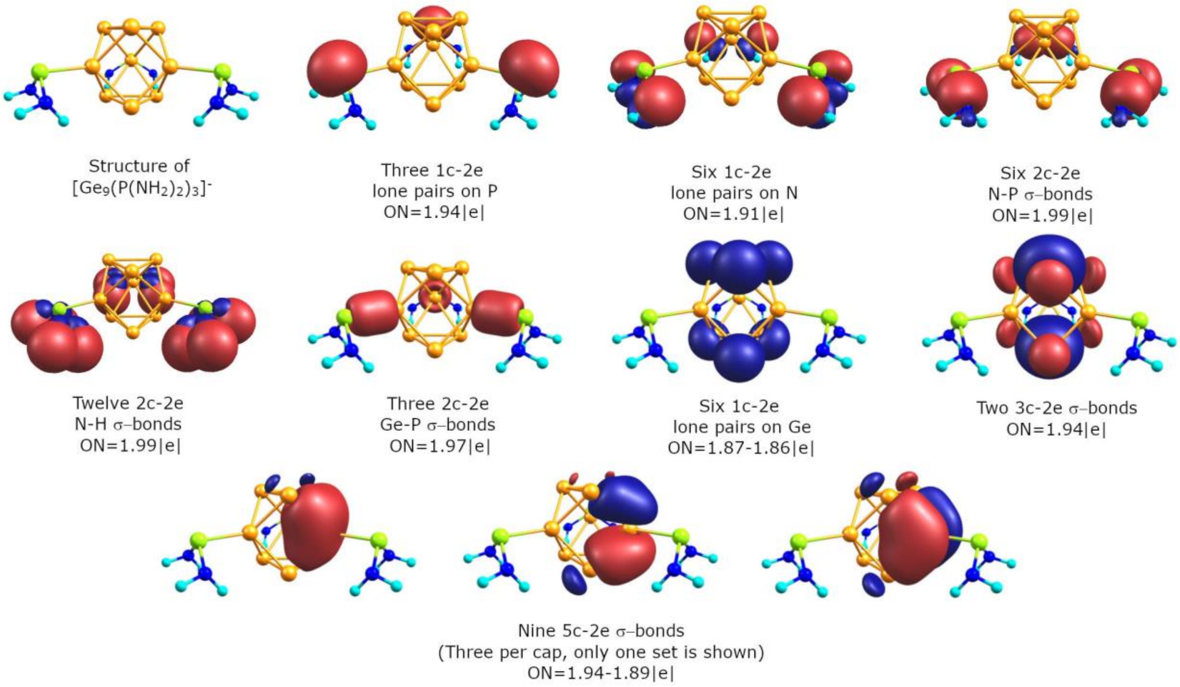

Figure S1. Chemical bonding picture of $[Ge_9\{P(NH_2)_2\}_3]^-$ cluster.

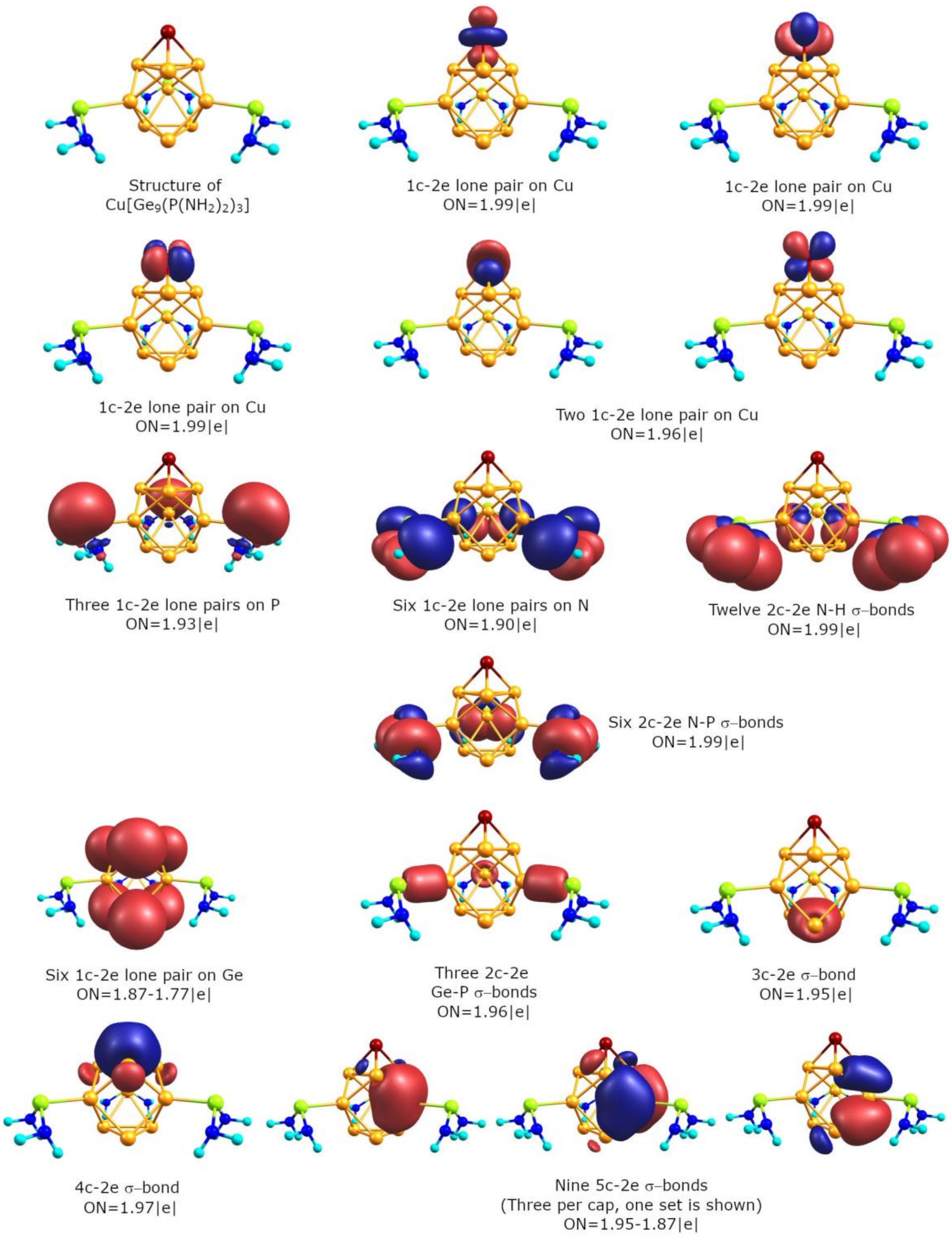

Figure S2. Chemical bonding picture of $Cu[Ge_9\{P(NH_2)_2\}_3]$ cluster.

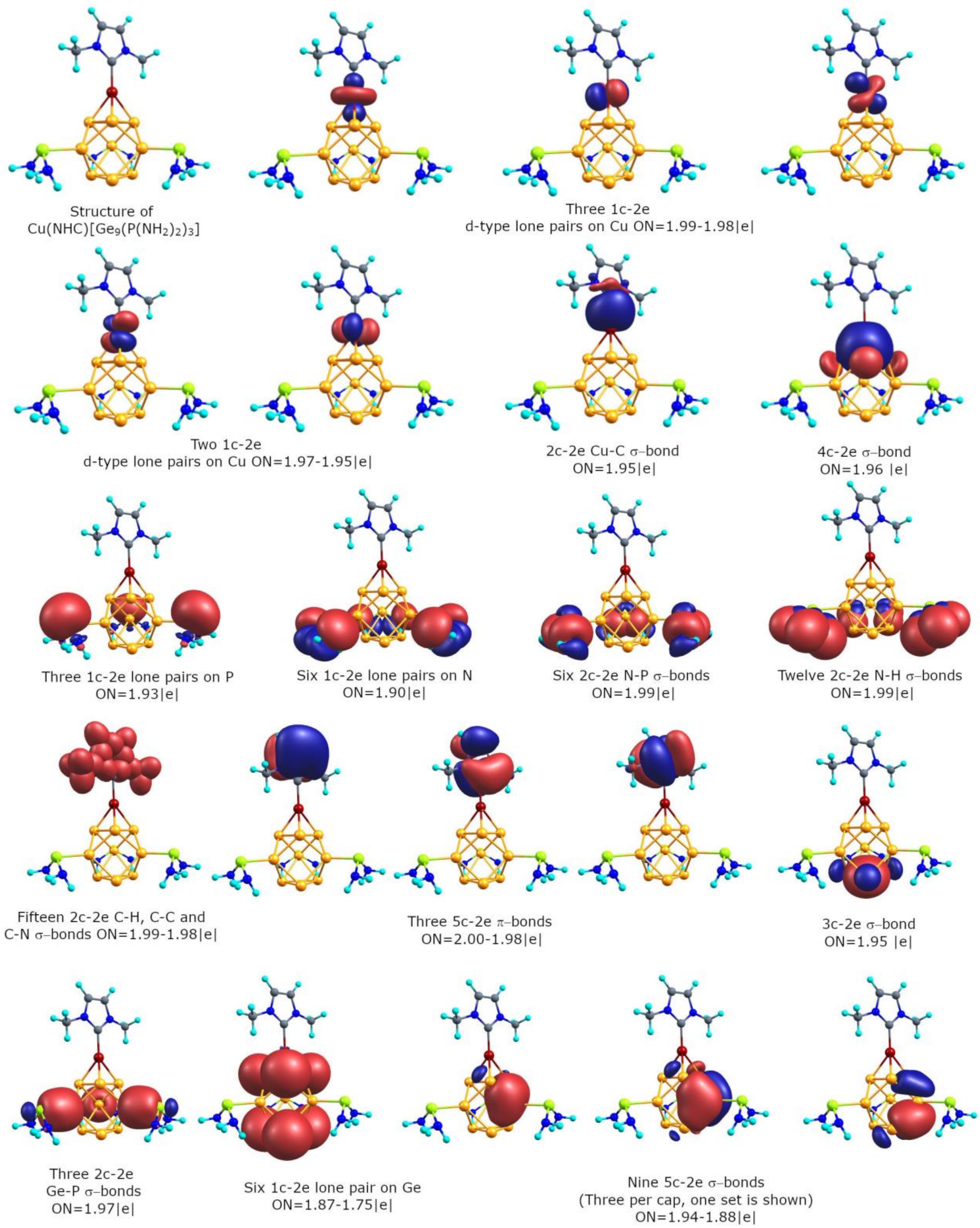

Figure S3. Chemical bonding picture of $Cu(NHC)[Ge_9\{P(NH_2)_2\}_3]$ cluster.

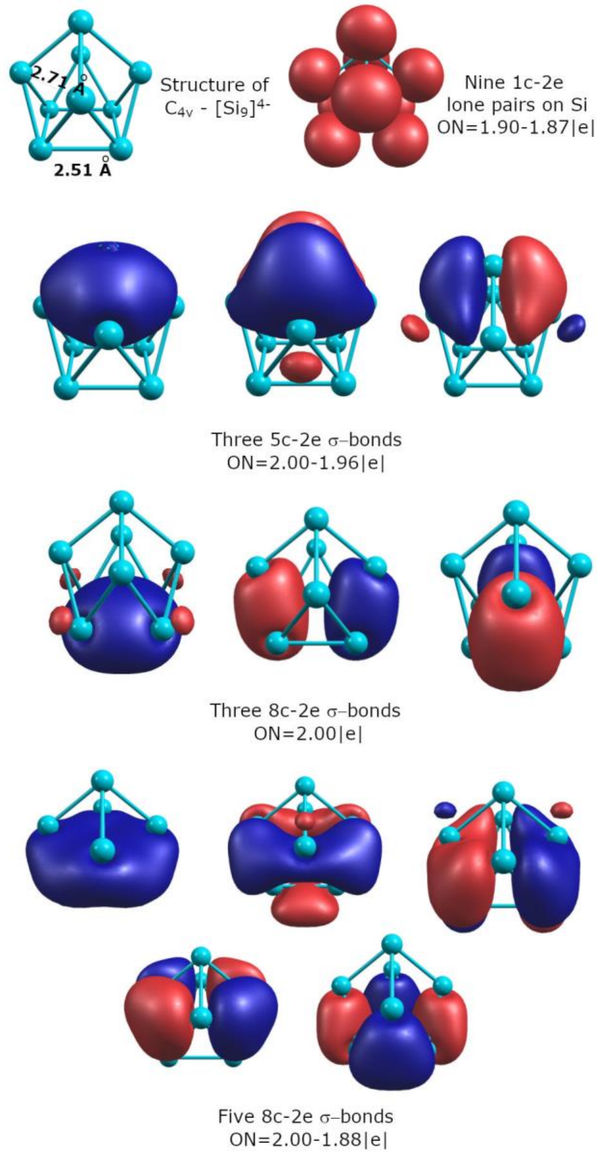

Figure S4. Chemical bonding picture of C_{4v} $[Si_9]^{4-}$ cluster.

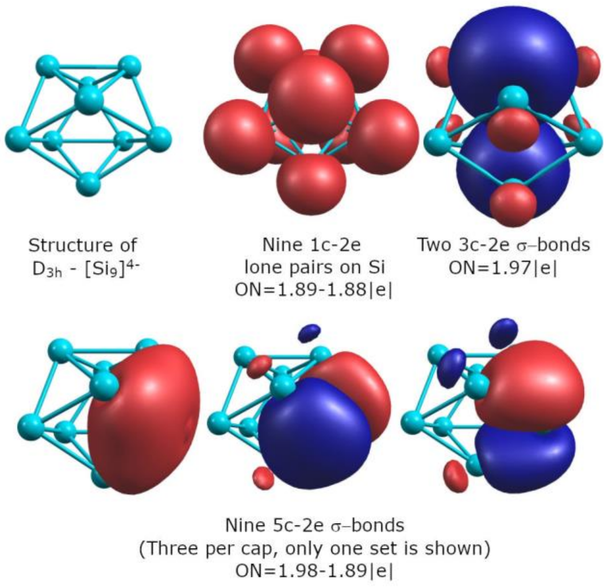

Figure S5. Chemical bonding picture of D_{3h} $[Si_9]^{4-}$ cluster.

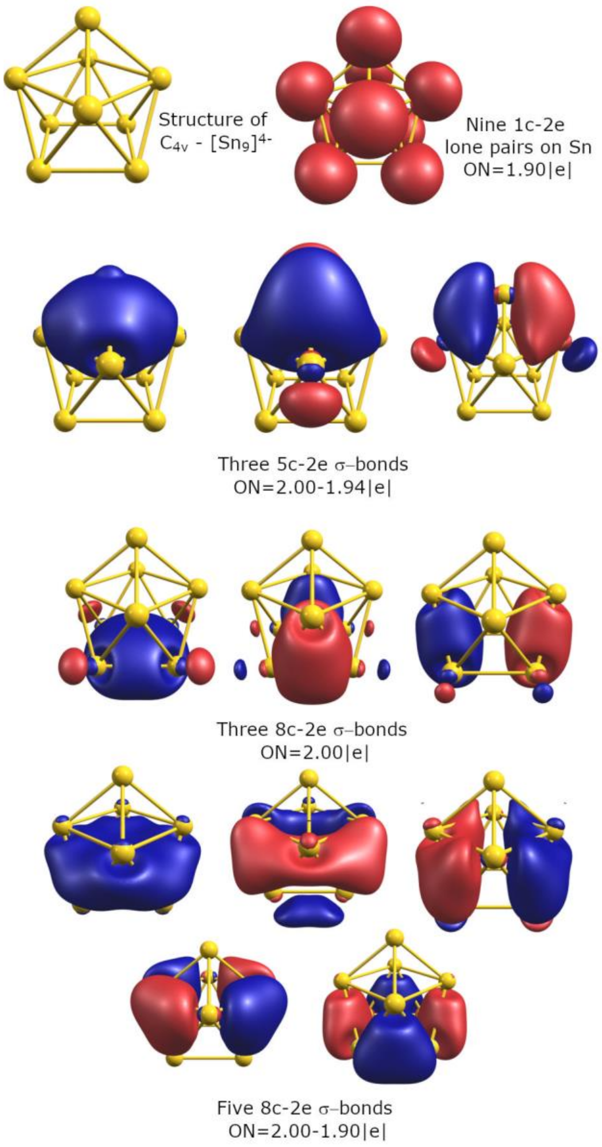

Figure S6. Chemical bonding picture of C_{4v} $[Sn_9]^{4-}$ cluster.

2. Optimized geometries of investigated molecules.

Table S1. Coordinates of optimized geometries, total energies and ZPE corrections of investigated molecules.

C_{4v} - $[Ge_9]^{4-}$	PBE0/LANL2DZ (0 imaginary Frequencies, E_{elec}= -33.6748928, ZPE correction= 0.006780)
	32 0.000000000 2.145834000 0.814083000<br><br>32 2.145834000 0.000000000 0.814083000

	32 1.377314000 1.377314000 -1.443071000<br><br>32 -2.145834000 0.000000000 0.814083000<br><br>32 -1.377314000 -1.377314000 -1.443071000<br><br>32 0.000000000 -2.145834000 0.814083000<br><br>32 -1.377314000 1.377314000 -1.443071000<br><br>32 0.000000000 0.000000000 2.515949000<br><br>32 1.377314000 -1.377314000 -1.443071000
D_{3h} - $[Ge_9]^{4-}$	PBE0/LANL2DZ (0 imaginary Frequencies, E_{elec}= -33.6748643, ZPE correction= 0.006748)
	32 -0.808114000 1.399561000 1.671373000<br><br>32 -0.808114000 -1.399716000 1.671129000<br><br>32 1.616349000 0.000069000 1.671003000<br><br>32 -0.808114000 1.399561000 -1.671373000<br><br>32 1.616349000 0.000069000 -1.671003000<br><br>32 -0.808114000 -1.399716000 -1.671129000<br><br>32 1.239488000 2.147435000 0.000000000<br><br>32 -2.479494000 0.000067000 0.000000000<br><br>32 1.239764000 -2.147329000 0.000000000
C_{4v} - $[Ge_9]^{4-}$	PBE0/aug-cc-pvdz (1 imaginary Frequency (-42.3924 cm^{-1}), E_{elec}= -18690.3069900, ZPE correction= 0.007066)
	32 2.031521000 0.000000000 0.789827000<br><br>32 0.000000000 -2.031521000 0.789827000<br><br>32 1.322067000 -1.322517000 -1.404647000<br><br>32 0.000000000 2.031521000 0.789827000<br><br>32 -1.322067000 1.322517000 -1.404647000<br><br>32 -2.031521000 0.000000000 0.789827000<br><br>32 1.322517000 1.322067000 -1.404647000

	32 0.000000000 0.000000000 2.459284000<br>32 -1.322517000 -1.322067000 -1.404647000
D_{3h} - $[Ge_9]^{4-}$	PBE0/ aug-cc-pvdz (0 imaginary Frequencies, E_{elec}= -18690.3096466, ZPE correction= 0.007200)
	32 0.791113000 1.370249000 1.502480000<br>32 -1.582227000 0.000000000 1.502480000<br>32 0.791113000 -1.370249000 1.502480000<br>32 0.791113000 1.370249000 -1.502480000<br>32 0.791113000 -1.370249000 -1.502480000<br>32 -1.582227000 0.000000000 -1.502480000<br>32 2.456815000 0.000124000 0.000000000<br>32 -1.228515000 2.127602000 0.000000000<br>32 -1.228300000 -2.127726000 0.000000000
C_{4v} - $[Ge_9]^{4-}$	PBE0/aug-cc-pvtz (1 imaginary Frequency (-123.5556 cm^{-1}), E_{elec}= -18690.9296291, ZPE correction= 0.006898)
	32 1.419027000 1.419014000 0.793758000<br>32 1.419014000 -1.419027000 0.793758000<br>32 1.857957000 0.000000000 -1.413427000<br>32 -1.419014000 1.419027000 0.793758000<br>32 -1.857957000 0.000000000 -1.413427000<br>32 -1.419027000 -1.419014000 0.793758000<br>32 0.000000000 1.857957000 -1.413427000<br>32 0.000000000 0.000000000 2.478676000<br>32 0.000000000 -1.857957000 -1.413427000
D_{3h} - $[Ge_9]^{4-}$	PBE0/ aug-cc-pvtz (0 imaginary Frequencies, E_{elec}= -18690.9465320, ZPE correction= 0.007440)
	32 -1.608451000 0.000005000 1.410505000

	32 0.804247000 -1.393009000 1.410273000<br>32 0.804247000 1.392987000 1.410320000<br>32 -1.608451000 0.000005000 -1.410505000<br>32 0.804247000 1.392987000 -1.410320000<br>32 0.804247000 -1.393009000 -1.410273000<br>32 -1.250522000 2.165909000 0.000000000<br>32 -1.250512000 -2.165864000 0.000000000<br>32 2.500947000 -0.000012000 0.000000000
C_{4v} - $[Ge_9]^{4-}$	B3LYP/LANL2DZ (0 imaginary Frequencies, E_{elec}= -33.5632179412, ZPE correction= 0.006303)
	32 1.536155000 1.536160000 0.820880000<br>32 1.536160000 -1.536155000 0.820880000<br>32 1.970253000 0.000000000 -1.454127000<br>32 -1.536160000 1.536155000 0.820880000<br>32 -1.970253000 0.000000000 -1.454127000<br>32 -1.536155000 -1.536160000 0.820880000<br>32 0.000000000 1.970253000 -1.454127000<br>32 0.000000000 0.000000000 2.532987000<br>32 0.000000000 -1.970253000 -1.454127000
D_{3h} - $[Ge_9]^{4-}$	B3LYP/LANL2DZ (0 imaginary Frequencies, E_{elec} = -33.5631968217, ZPE correction= 0.006273)
	32 -0.815929000 1.413230000 1.693468000<br>32 -0.815929000 -1.413230000 1.693468000<br>32 1.631858000 0.000000000 1.693468000<br>32 -0.815929000 1.413230000 -1.693468000<br>32 1.631858000 0.000000000 -1.693468000<br>32 -0.815929000 -1.413230000 -1.693468000

	32 1.251034000 2.166937000 0.000000000<br>32 -2.502139000 -0.000041000 0.000000000<br>32 1.251105000 -2.166895000 0.000000000
C_{4v} - $[Ge_9]^{4-}$	B3LYP/aug-cc-pvdz (1 imaginary Frequency (-18.8437 cm^{-1}), E_{elec}= -18693.0103353, ZPE correction= 0.006405)
	32 1.472724000 1.472709000 0.801639000<br>32 1.472709000 -1.472724000 0.801639000<br>32 1.909141000 0.000000000 -1.422199000<br>32 -1.472709000 1.472724000 0.801639000<br>32 -1.909141000 0.000000000 -1.422199000<br>32 -1.472724000 -1.472709000 0.801639000<br>32 0.000000000 1.909141000 -1.422199000<br>32 0.000000000 0.000000000 2.482237000<br>32 0.000000000 -1.909141000 -1.422199000
D_{3h} - $[Ge_9]^{4-}$	B3LYP/aug-cc-pvdz (0 imaginary Frequencies, E_{elec} = -18693.0109539, ZPE correction= 0.006433)
	32 1.601809000 -0.000051000 1.576911000<br>32 -0.800922000 1.387309000 1.576725000<br>32 -0.800922000 -1.387194000 1.576915000<br>32 1.601809000 -0.000051000 -1.576911000<br>32 -0.800922000 -1.387194000 -1.576915000<br>32 -0.800922000 1.387309000 -1.576725000<br>32 1.233353000 -2.136376000 0.000000000<br>32 1.233544000 2.136261000 0.000000000<br>32 -2.466828000 -0.000013000 0.000000000
C_{4v} - $[Ge_9]^{4-}$	B3LYP/aug-cc-pvtz (1 imaginary Frequency (-129.9552 cm^{-1}), E_{elec}= -18693.6362612, ZPE correction= 0.006211)

	32 0.000000000 2.051750000 0.805061000<br>32 2.051750000 0.000000000 0.805061000<br>32 1.337526000 1.337526000 -1.430326000<br>32 -2.051750000 0.000000000 0.805061000<br>32 -1.337526000 -1.337526000 -1.430326000<br>32 0.000000000 -2.051750000 0.805061000<br>32 -1.337526000 1.337526000 -1.430326000<br>32 0.000000000 0.000000000 2.501061000<br>32 1.337526000 -1.337526000 -1.430326000
D_{3h} - $[Ge_9]^{4-}$	B3LYP/aug-cc-pvtz (0 imaginary Frequencies, E_{elec} = -18693.6512336, ZPE correction= 0.006719)
	32 -0.822647000 1.424872000 1.434270000<br>32 -0.822647000 1.424872000 -1.434270000<br>32 -2.525391000 0.000000000 0.000000000<br>32 1.645296000 0.000000000 1.434394000<br>32 1.262693000 -2.186984000 0.000000000<br>32 1.645296000 0.000000000 -1.434394000<br>32 -0.822647000 -1.424872000 1.434271000<br>32 1.262693000 2.186984000 0.000000000<br>32 -0.822647000 -1.424872000 -1.434271000
$[Ge_9(P(NH_2)_2)_3]^-$	PBE0/ aug-cc-pvdz (2 degenerate imaginary Frequencies (rotation of -$P(NH_2)_2$ groups, -11.8015 cm^{-1}), E_{elec}= -20049.9555733, ZPE correction= 0.169815)
	32 1.359718000 0.785033000 1.924924000<br>32 -1.359718000 0.785033000 1.924924000<br>32 0.000000000 -1.570067000 1.924924000<br>32 1.342156000 0.774894000 -1.584128000

	32 0.000000000 -1.549788000 -1.584128000<br>32 -1.342156000 0.774894000 -1.584128000<br>32 1.779103000 -1.027166000 0.187338000<br>32 0.000000000 2.054332000 0.187338000<br>32 -1.779103000 -1.027166000 0.187338000<br>15 0.000000000 4.396758000 0.087242000<br>7 1.437965000 4.563066000 -0.895191000<br>1 1.543398000 3.946082000 -1.696258000<br>1 1.656366000 5.522403000 -1.141854000<br>7 -1.437965000 4.563066000 -0.895191000<br>1 -1.656366000 5.522403000 -1.141854000<br>1 -1.543398000 3.946082000 -1.696258000<br>15 3.807705000 -2.198379000 0.087242000<br>7 3.232748000 -3.526847000 -0.895191000<br>1 2.645708000 -3.309663000 -1.696258000<br>1 3.954358000 -4.195656000 -1.141854000<br>7 4.670713000 -1.036219000 -0.895191000<br>1 5.610724000 -1.326747000 -1.141854000<br>1 4.189106000 -0.636419000 -1.696258000<br>15 -3.807705000 -2.198379000 0.087242000<br>7 -3.232748000 -3.526847000 -0.895191000<br>1 -3.954358000 -4.195656000 -1.141854000<br>1 -2.645708000 -3.309663000 -1.696258000<br>7 -4.670713000 -1.036219000 -0.895191000<br>1 -4.189106000 -0.636419000 -1.696258000<br>1 -5.610724000 -1.326747000 -1.141854000

$[Ge_9(P(NH_2)_2)_3]^-$	PBE0/LANL2DZ (0 imaginary Frequencies, E_{elec}= -389.038813956, ZPE correction= 0.171352)
	32 1.437706000 0.830060000 1.968781000<br>32 -1.437706000 0.830060000 1.968781000<br>32 0.000000000 -1.660119000 1.968781000<br>32 1.417056000 0.818138000 -1.637792000<br>32 0.000000000 -1.636275000 -1.637792000<br>32 -1.417056000 0.818138000 -1.637792000<br>32 1.830812000 -1.057020000 0.179480000<br>32 0.000000000 2.114040000 0.179480000<br>32 -1.830812000 -1.057020000 0.179480000<br>15 0.000000000 4.525417000 0.102796000<br>7 1.417310000 4.738741000 -0.927912000<br>1 1.718004000 4.068239000 -1.623332000<br>1 1.987586000 5.564738000 -0.819768000<br>7 -1.417310000 4.738741000 -0.927912000<br>1 -1.987586000 5.564738000 -0.819768000<br>1 -1.718004000 4.068239000 -1.623332000<br>15 3.919126000 -2.262708000 0.102796000<br>7 3.395215000 -3.596797000 -0.927912000<br>1 2.664196000 -3.521955000 -1.623332000<br>1 3.825411000 -4.503669000 -0.819768000<br>7 4.812525000 -1.141944000 -0.927912000<br>1 5.812998000 -1.061069000 -0.819768000<br>1 4.382201000 -0.546284000 -1.623332000<br>15 -3.919126000 -2.262708000 0.102796000

	7 -3.395215000 -3.596797000 -0.927912000<br>1 -3.825411000 -4.503669000 -0.819768000<br>1 -2.664196000 -3.521955000 -1.623332000<br>7 -4.812525000 -1.141944000 -0.927912000<br>1 -4.382201000 -0.546284000 -1.623332000<br>1 -5.812998000 -1.061069000 -0.819768000
Cu[$Ge_9(P(NH_2)_2)_3$]	PBE0/aug-cc-pvdz (0 imaginary Frequencies, E_{elec}= -21690.2344825, ZPE correction= 0.171381)
	32 -1.832411000 -1.524237000 0.000000000<br>32 0.915843000 -1.525714000 -1.586340000<br>32 0.915843000 -1.525714000 1.586340000<br>32 -1.525858000 1.819144000 0.000000000<br>32 0.762526000 1.820033000 1.320857000<br>32 0.762526000 1.820033000 -1.320857000<br>32 -1.047006000 0.002151000 1.814481000<br>32 -1.047006000 0.002151000 -1.814481000<br>32 2.094135000 0.001998000 0.000000000<br>15 -2.217161000 0.053768000 -3.841491000<br>7 -3.550553000 1.018891000 -3.287048000<br>1 -3.368867000 1.909562000 -2.835208000<br>1 -4.308501000 1.097390000 -3.955883000<br>7 -1.066319000 1.012624000 -4.720766000<br>1 -1.266239000 1.089902000 -5.711760000<br>1 -0.762763000 1.903025000 -4.339280000<br>15 -2.217161000 0.053768000 3.841491000<br>7 -1.066319000 1.012624000 4.720766000

	1 -0.762763000 1.903025000 4.339280000<br>1 -1.266239000 1.089902000 5.711760000<br>7 -3.550553000 1.018891000 3.287048000<br>1 -4.308501000 1.097390000 3.955883000<br>1 -3.368867000 1.909562000 2.835208000<br>15 4.434631000 0.053933000 0.000000000<br>7 4.620662000 1.015870000 1.434219000<br>1 5.578837000 1.094309000 1.756300000<br>1 4.137761000 1.905953000 1.505161000<br>7 4.620662000 1.015870000 -1.434219000<br>1 4.137761000 1.905953000 -1.505161000<br>1 5.578837000 1.094309000 -1.756300000<br>29 -0.001142000 -3.157266000 0.000000000
Cu(NHC)[$Ge_9(P(NH_2)_2)_3$]	PBE0/aug-cc-pvdz (0 imaginary Frequencies, E_{elec}= -21994.7799089, ZPE correction= 0.300350)
	32 0.857327000 0.820130000 -1.477631000<br>32 0.856931000 0.864125000 1.450580000<br>32 0.822825000 -1.694321000 0.023991000<br>32 -2.566494000 0.761778000 -1.338993000<br>32 -2.565595000 -1.517848000 0.023398000<br>32 -2.563176000 0.801571000 1.317863000<br>32 -0.772535000 -1.052814000 -1.793477000<br>32 -0.755714000 2.078736000 -0.032481000<br>32 -0.770432000 -1.000388000 1.823657000<br>15 -0.745359000 4.417255000 -0.064860000<br>7 -1.704877000 4.613442000 -1.503010000

	1 -2.587304000 4.116812000 -1.579312000<br>1 -1.802711000 5.576594000 -1.804579000<br>7 -1.701575000 4.653473000 1.369472000<br>1 -1.799227000 5.624731000 1.643900000<br>1 -2.583623000 4.158850000 1.461638000<br>15 -0.796980000 -2.275113000 -3.787313000<br>7 -1.742966000 -3.613260000 -3.202164000<br>1 -2.617730000 -3.426687000 -2.721335000<br>1 -1.850945000 -4.364848000 -3.874264000<br>7 -1.775481000 -1.163465000 -4.700374000<br>1 -1.885852000 -1.417750000 -5.675700000<br>1 -2.653569000 -0.839069000 -4.306979000<br>15 -0.793480000 -2.160187000 3.854532000<br>7 -1.744081000 -3.513210000 3.312706000<br>1 -1.852545000 -4.243480000 4.007836000<br>1 -2.619462000 -3.339072000 2.828349000<br>7 -1.767362000 -1.018473000 4.734813000<br>1 -2.645620000 -0.704388000 4.333505000<br>1 -1.876025000 -1.242124000 5.717807000<br>29 2.733465000 -0.025586000 -0.000610000<br>6 4.700274000 -0.023220000 -0.000173000<br>7 5.549359000 -1.081642000 0.000878000<br>6 6.865137000 -0.673636000 0.000318000<br>6 6.847118000 0.685325000 -0.001361000<br>7 5.520983000 1.057507000 -0.001290000<br>1 7.695455000 -1.369301000 0.001306000

	1 7.658702000 1.402737000 -0.002295000<br><br>6 5.123837000 -2.467599000 0.001885000<br><br>1 5.504613000 -2.979523000 0.893895000<br><br>1 4.029489000 -2.491057000 0.010527000<br><br>1 5.490371000 -2.976943000 -0.897583000<br><br>6 5.056142000 2.430674000 -0.003893000<br><br>1 5.408830000 2.948986000 -0.903648000<br><br>1 3.961980000 2.422019000 0.002106000<br><br>1 5.418513000 2.955310000 0.888268000
$\{Cu[Ge_9\{P(NH_2)_2\}_3]_2\}^-$	PBE0/aug-cc-pvdz (0 imaginary Frequencies, E_{elec}= -41740.2521030, ZPE correction= 0.341278)
	32 -1.971214000 -1.152141000 -1.224550000<br><br>32 -1.969346000 1.630427000 -0.392466000<br><br>32 -1.967227000 -0.482928000 1.602278000<br><br>32 -5.418742000 -1.053371000 -1.113708000<br><br>32 -5.408335000 -0.439755000 1.476214000<br><br>32 -5.412880000 1.496423000 -0.350742000<br><br>32 -3.614915000 -2.009963000 0.472926000<br><br>32 -3.619490000 0.590070000 -1.978850000<br><br>32 -3.609580000 1.417595000 1.500758000<br><br>15 -3.719878000 1.264191000 -4.218769000<br><br>7 -4.702972000 -0.061273000 -4.784673000<br><br>1 -5.535730000 -0.311560000 -4.259463000<br><br>1 -4.891173000 -0.037709000 -5.780679000<br><br>7 -4.692634000 2.688657000 -3.958213000<br><br>1 -4.878365000 3.219906000 -4.801575000

	1	-5.524698000	2.613900000	-3.380127000
	15	-3.711111000	-4.284855000	1.018202000
	7	-4.676581000	-4.107123000	2.460185000
	1	-5.508994000	-3.525959000	2.423315000
	1	-4.859349000	-4.979635000	2.943176000
	7	-4.700665000	-4.774197000	-0.332697000
	1	-4.888390000	-5.770040000	-0.365492000
	1	-5.534778000	-4.235883000	-0.548350000
	15	-3.700004000	3.022004000	3.203426000
	7	-4.673196000	2.088038000	4.309726000
	1	-4.855171000	2.553989000	5.191642000
	1	-5.507807000	1.627635000	3.958268000
	7	-4.681092000	4.177648000	2.340242000
	1	-5.516077000	3.851154000	1.862536000
	1	-4.864231000	5.030006000	2.857768000
	29	0.000031000	-0.002229000	-0.007930000
	32	1.969367000	-1.633628000	0.380613000
	32	1.967452000	1.149452000	1.215651000
	32	1.971364000	0.479684000	-1.611072000
	32	5.413882000	-1.494335000	0.357465000
	32	5.418376000	0.442300000	-1.468105000
	32	5.408008000	1.055446000	1.122155000
	32	3.619878000	-1.415739000	-1.503481000
	32	3.610096000	-0.594802000	1.976939000
	32	3.614528000	2.008449000	-0.478929000
	15	3.700787000	-1.262764000	4.219091000

	7 4.684947000 -2.680932000 3.967424000<br>1 5.519929000 -2.601658000 3.394284000<br>1 4.869323000 -3.209164000 4.812942000<br>7 4.670835000 0.070573000 4.788331000<br>1 4.851664000 0.051704000 5.785750000<br>1 5.505090000 0.326321000 4.268023000<br>15 3.720331000 -3.021939000 -3.203939000<br>7 4.703990000 -2.091069000 -4.303470000<br>1 5.536518000 -1.631559000 -3.946047000<br>1 4.891984000 -2.558063000 -5.183514000<br>7 4.692314000 -4.179004000 -2.332163000<br>1 4.878226000 -5.031775000 -2.848146000<br>1 5.524243000 -3.853320000 -1.848512000<br>15 3.708909000 4.286202000 -1.012143000<br>7 4.696047000 4.118413000 -2.440720000<br>1 4.881951000 4.993715000 -2.917400000<br>1 5.530667000 3.541133000 -2.393899000<br>7 4.676384000 4.774241000 0.354846000<br>1 5.509307000 4.238500000 0.581331000<br>1 4.858722000 5.770737000 0.395311000

3. Coordinates of chosen points for NICS calculation.

Table S2. Coordinates of points for NICS calculation.

D_{3h} - $[Ge_9]^{4-}$	32 1.608480000 0.000000000 -1.410366000<br>32 -0.804240000 -1.392985000 -1.410366000<br>32 -0.804240000 1.392985000 -1.410366000

	32 1.608480000 0.000000000 1.410366000<br>32 -0.804240000 1.392985000 1.410366000<br>32 -0.804240000 -1.392985000 1.410366000<br>32 1.250482000 2.165898000 0.000000000<br>32 1.250482000 -2.165898000 0.000000000<br>32 -2.500963000 0.000000000 0.000000000<br>Point 1 -1.652600000 0.000000000 0.000000000<br>Point 2 -0.804240000 0.000000000 0.000000000<br>Point 3 0.000000000 0.000000000 0.000000000<br>Point 4 0.000000000 0.000000000 -1.410366000<br>Point 5 0.000000000 0.000000000 -2.410366000
C_{4v} - $[Ge_9]^{4-}$	32 1.419021000 1.419021000 -0.793758000<br>32 -1.419021000 1.419021000 -0.793758000<br>32 0.000000000 1.857957000 1.413427000<br>32 1.419021000 -1.419021000 -0.793758000<br>32 0.000000000 -1.857957000 1.413427000<br>32 -1.419021000 -1.419021000 -0.793758000<br>32 1.857957000 0.000000000 1.413427000<br>32 0.000000000 0.000000000 -2.478676000<br>32 -1.857957000 0.000000000 1.413427000<br>Point 1 0.000000000 0.000000000 -1.636218000<br>Point 2 0.000000000 0.000000000 -0.793758000<br>Point 3 0.000000000 0.000000000 0.309837000<br>Point 4 0.000002000 0.000000000 1.413427000<br>Point 5 0.000002000 0.000000000 2.413427000
